# Eosinophil ETosis in severe non-IgE-mediated gastrointestinal food allergy, a neonatal necrotizing enterocolitis: A case report

**DOI:** 10.5415/apallergy.0000000000000213

**Published:** 2025-12-02

**Authors:** Aya Imaide, Shoichiro Taniuchi, Shuhei Dohi, Masatoshi Mitomori, Meguru Gouma, Masahiro Enomoto, Masamitsu Nishino, Yoshiyuki Yamada, Yuki Moritoki, Shigeharu Ueki, Yo Okizuka

**Affiliations:** 1 Department of Pediatrics, Takatsuki General Hospital, Osaka, Japan; 2 Department of Pediatrics, Chibune General Hospital, Osaka, Japan; 3 Department of Pediatrics, Tokai University School of Medicine, Isehara, Japan; 4 Department of General Internal Medicine and Clinical Laboratory Medicine, Akita University Graduate School of Medicine, Akita, Japan

**Keywords:** Case report, Charcot–Leyden crystals, ETosis, extracellular traps, non-IgE-mediated gastrointestinal food allergy

## Abstract

Non-IgE-mediated gastrointestinal food allergy is a hypersensitivity disorder that affects the gastrointestinal tract, operating independently of IgE. While eosinophilic inflammation has been implicated in the literature, its precise role in the pathophysiology of non-IgE-mediated gastrointestinal food allergy remains poorly understood. We report the first documented case of severe non-IgE-mediated gastrointestinal food allergy in which eosinophil infiltration, eosinophil extracellular traps, and Charcot–Lyden crystals were observed in the tissue. A 1-month-old female infant presented with fever, watery diarrhea, and poor weight gain associated with the ingestion of cow’s milk-based formula. Despite transitioning to an extensively hydrolyzed formula, she developed necrotizing enterocolitis. Following a switch to an amino-acid-based infant formula, her abdominal symptoms resolved, and her weight gain normalized. However, 1 month later, bile-like vomiting led to the diagnosis of jejunal atresia, which was attributed to scarring from necrotizing enterocolitis. Surgical resection revealed histopathological findings of eosinophil infiltration, cytolytic eosinophil cell death (ETosis), extracellular traps, and Charcot–Lyden crystals. The necrotizing enterocolitis was ultimately diagnosed as a severe manifestation of non-IgE-mediated gastrointestinal food allergy triggered by cow’s milk protein. After surgery, the patient resumed feeding with an amino-acid-based infant formula. During regular follow-up, she continued to gain weight well without any clinical symptoms while being fed an amino-acid-based infant formula. To our knowledge, this is the first report to identify a histopathological link between severe non-IgE-mediated gastrointestinal food allergy-related inflammation and eosinophil ETosis. This suggests a potential role of ETosis in the pathogenesis and its complications.

## 1. Introduction

Non-IgE-mediated gastrointestinal food allergy (non-IgE-GIFA), including food protein-induced enterocolitis syndrome (FPIES), is characterized by gastrointestinal symptoms in the absence of IgE-mediated hypersensitivity [1]. While eosinophil inflammation has been noted in some non-IgE-GIFA cases, its pathophysiology remains poorly understood [2]. Recent studies have identified the mechanism of eosinophil cell death (ETosis), along with the subsequent release of net-like chromatin fibers (eosinophil extracellular traps [EETs]), granule proteins, and the formation of Charcot–Lyden crystals [3]. Here, we report the first documented case of severe non-IgE-GIFA in which eosinophil infiltration, EETs, and Charcot–Lyden crystals were observed in the tissue.

## 2. Case report

A female infant was born at 38 weeks of gestation with a birth weight of 4056 g. Her prenatal, neonatal, and family history were unremarkable. She was fed cow’s milk-based formula after birth and developed watery stools and poor weight gain from 13 days of age. At 28 days of age, she was hospitalized at a community hospital for fever and frequent diarrhea. Laboratory tests showed increased C-reactive protein (14.4 mg/dL) and white blood cell count (15,580/μL, with eosinophils at 93/μL). A comprehensive investigation, including sepsis evaluation, was conducted, but the cause remained unclear. Elevated cow’s milk-specific IgE and casein-specific IgE (1.2 and 1.0 UA/mL, respectively) led to a presumptive diagnosis of cow’s milk allergy, and an extensively hydrolyzed formula (eHF) was introduced at 40 days of age.

**Figure 1.**
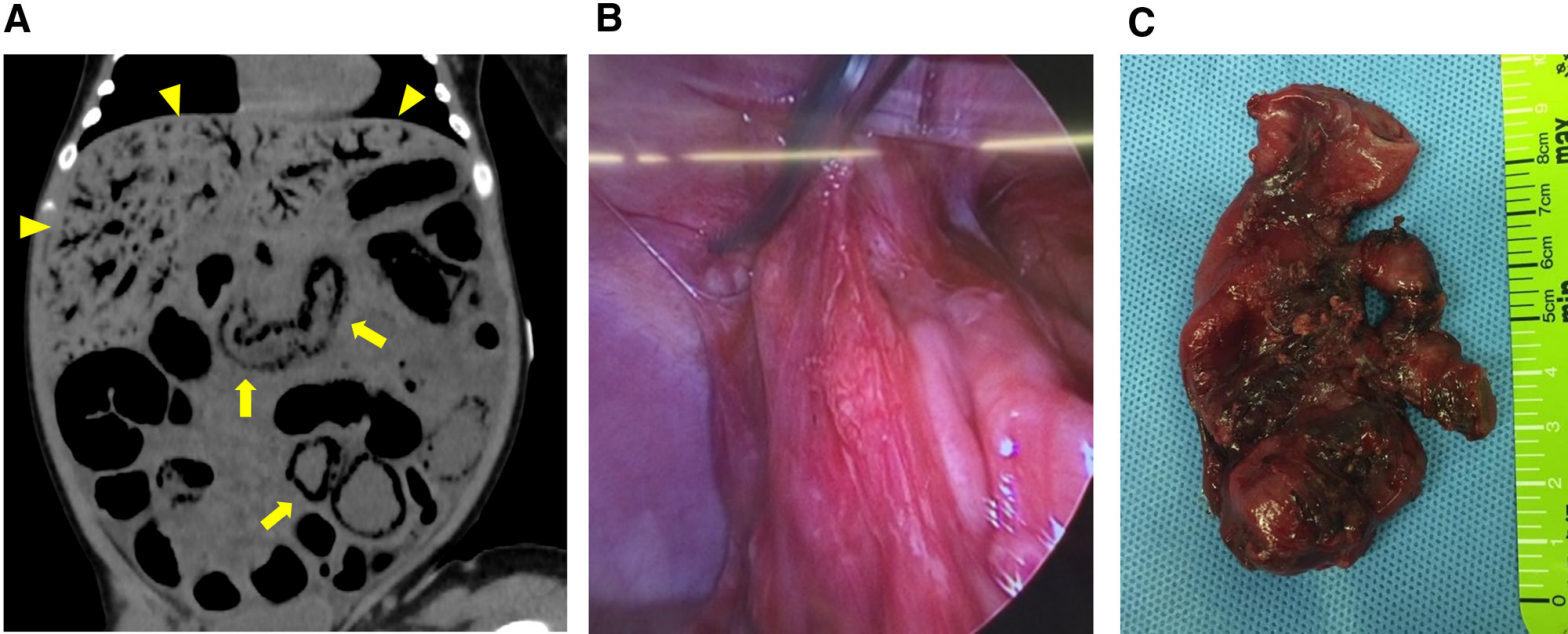
(A) Abdominal computed tomography at 49 days of age revealed portal venous gas (arrowhead) and intestinal dilatation with pneumatosis intestinalis (arrows), indicative necrotizing enterocolitis. (B) Intraoperative findings at 116 days of age showed dense adhesions of the jejunum due to scarring from necrotizing enterocolitis. (C) Resected jejunal specimen.

At 49 days of age, the patient presented in shock with fever, vomiting, abdominal distension, and bloody stools. She was transferred to the Pediatric Intensive Care Unit of our hospital. Laboratory findings revealed increased C-reactive protein (14.2 mg/dL), white blood cell count (15,200/μL; no eosinophils), and metabolic acidosis. Abdominal computed tomography showed portal venous gas and pneumatosis intestinalis, consistent with a diagnosis of necrotizing enterocolitis (NEC) (Fig. 1). Supportive treatment, including bowel rest, was instituted. Given the suspicion that NEC was caused by cow’s milk allergy to eHF, an amino-acid-based formula (AAP) was introduced at 63 days of age after her condition stabilized. Subsequent clinical course was favorable, with a resolution of gastrointestinal symptoms and catch-up weight gain.

Approximately 1 month later, the patient developed bile-like vomiting, which progressively worsened. Upper gastrointestinal series demonstrated jejunal atresia and surgical resection was performed. The atresia was attributed to scarring secondary to inflammation caused by NEC.

Histopathological examination of the resected tissue with hematoxylin and eosin staining revealed multiple ulcers and infiltration of inflammatory cells, including a large number of eosinophils (>20/high power field) in the jejunum mucosa. Based on these histopathological findings and the clinical course, the patient was considered to have severe non-IgE-GIFA.

**Figure 2.**
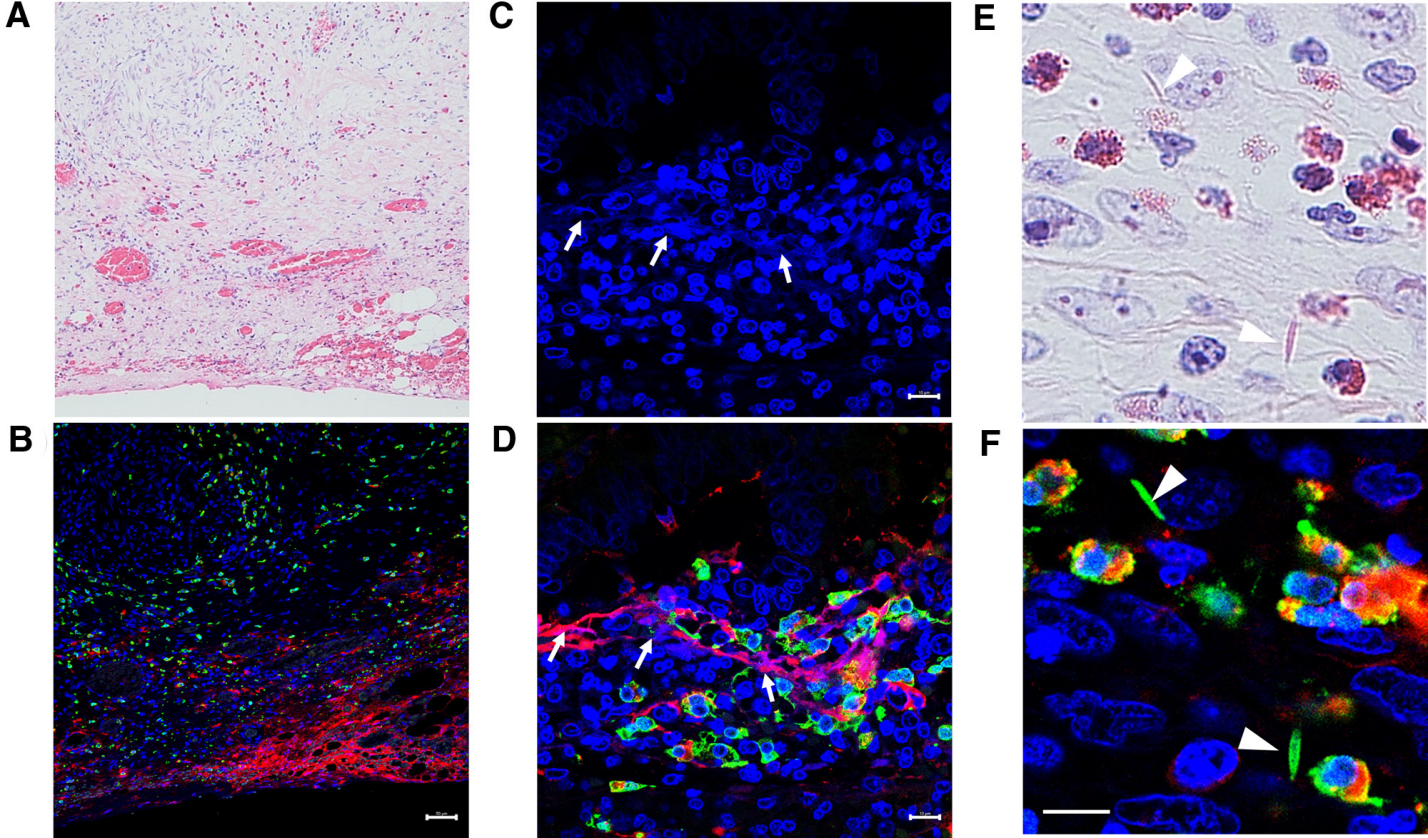
Histopathological findings of the resected tissue. Tissue sections were subjected to immunofluorescence staining for galectin-10 (green), major basic protein (MBP; red), and DNA (blue), followed by counterstaining with hematoxylin and eosin (H&E). (A) H&E-stained tissue section (×200 magnification) showing extensive eosinophilic infiltration. (B) Corresponding immunofluorescence image (scale bar: 50 µm). (C) Higher magnification image of DNA staining and (D) the corresponding merged immunofluorescence image showing galectin-10 and MBP. Net-like extracellular DNA structures (arrows) were observed in close proximity to lytic eosinophils and tissue-deposited MBP and did not colocalize with intact galectin-10-positive eosinophils (×1000 magnification, scale bar: 10 µm). (E, F) Needle-like structures were positive for galectin-10, confirming their identity as Charcot–Leyden crystals (arrowheads, ×1000 magnification, scale bar: 10 µm).

To further evaluate eosinophilic inflammation, immunofluorescence staining for major basic protein and galectin-10, which is a cytoplasmic protein that forms Charcot–Lyden crystals, was performed following the previously established method (Fig. 2) [4]. Extensive major basic protein deposition was observed even in areas where eosinophils appeared absent on conventional hematoxylin and eosin staining. The presence of net-like DNA from disrupted eosinophils, forming EETs, and Charcot–Lyden crystals were identified.

After surgery, the patient resumed feeding with AAP and was discharged at 180 days of age. The diagnosis was NEC as a result of non-IgE-GIFA triggered by cow’s milk protein. During regular follow-up, she continued to gain weight well without any clinical symptoms while being fed AAP.

## 3. Discussion

The present case illustrated a severe form of non-IgE-GIFA, complicated by NEC and jejunal atresia. Although the clinical features partially met the criteria for chronic FPIES [5], deviations were observed. Chronic FPIES is typically defined by the resolution of symptoms within days following the elimination of the causative food and the acute recurrence of symptoms upon re-exposure. However, in our case, it required 2 weeks after food elimination before enteral nutrition could be resumed, and symptom recurrence was observed 1 week after initiation of eHF. These deviations may be attributable to the severe illness due to NEC, which likely prolonged the recovery period, and to the use of a low-allergen formula, which may have attenuated symptom provocation. Taken together, these findings suggest a clinical course overlapping with that of chronic FPIES. Furthermore, while our patient demonstrated elevated milk- and casein-specific IgE, similar findings have been reported in FPIES cases [5, 6]. In the absence of cutaneous or respiratory symptoms, a diagnosis of non-IgE-GIFA remains appropriate.

Eosinophilic inflammation in non-IgE-GIFA, including FPIES, has been previously described. Infiltration of eosinophils beyond normal levels into the intestinal mucosa of early infants with milk-induced FPIES has been reported in several reports [6–9]. In our case, marked eosinophilic infiltration was confirmed in the jejunal resection specimen, supporting eosinophil involvement.

ETosis is a cytolytic process in eosinophils caused by cell death resulting from excessive activation of the cells [3]. During this process, cell-free granules and net-like chromatin fiber, referred to as EETs, are released. The granules released contain specific proteins, including major basic protein, which exhibit cytotoxic effects not only against external pathogens but also on host tissues. Furthermore, when eosinophils rapidly rupture their plasma membranes via ETosis, galectin-10 is released into the extracellular environment, forming Charcot–Lyden crystals. EETs and Charcot–Lyden crystals persist in tissues for a certain period, contributing to the enhancement and prolongation of inflammation [10]. The contribution of eosinophil ETosis has been reported in various allergic disorders, such as eosinophilic chronic rhinosinusitis, where it is implicated in chronic inflammation and disease exacerbation [11]. It is thought to contribute to chronic inflammation and disease exacerbation through these mechanisms. However, to date, there have been no reports describing its involvement in non-IgE-GIFA. In our case, extracellular granule protein deposition and net-like DNA were observed in the mucosa of the resected tissue, confirming the presence of EETs in non-IgE-GIFA. These findings suggest that eosinophilic inflammation with eosinophil ETosis may be involved in the pathogenesis of NEC. However, since pathological findings from the acute phase have not been obtained, additional cases need to validate this hypothesis.

To our knowledge, this is the first report demonstrating a histopathological association between severe non-IgE-GIFA inflammation and eosinophil ETosis. This case supports a potential role for eosinophil ETosis in mediating severe inflammation in non-IgE-GIFA.

## Acknowledgements

The authors thank Ms. Noriko Tan for her exceptional technical assistance. We also acknowledge proofreading and editing by Benjamin Phillis.

This study was supported by JSPS KAKENHI (21K07833 and 24K11593 to SU), Research Grants on Allergic Disease and Immunology from The Japan Agency for Medical Research and Development (JP22ek0410097 and MHLW 202213003A to SU).

## Conflicts of interest

SU has received grants and personal fees from AstraZeneca, GlaxoSmithKline, and Sanofi and grants from Novartis, VIB, and Crytrill. The remaining authors declare no conflicts of interest.

## Author contributions

AI, ST, SD, MM, MG, ME, and YO were involved in the clinical care of the patient, including diagnosis, treatment, and follow-up. They also collected the clinical data and drafted the manuscript. MN provided consultation and contributed to the interpretation of clinical findings and manuscript review. YY supported the histopathological analysis, contributed to the diagnosis, and assisted in the literature review and critical revision of the manuscript. YM and SU performed immunofluorescence staining for major basic protein, galectin-10, and net-like DNA. They also contributed to the discussion, literature review, and critical revision of the manuscript.
